# Development of a mutation hotspot detection kit for the phenylalanine hydroxylase gene by ARMS-PCR combined with fluorescent probe technology

**DOI:** 10.1042/BSR20201660

**Published:** 2021-02-19

**Authors:** Rong Qiang, Lin Wang, JinHua He, Wei Jie Xu, Wei Li, Na Cai, Xiao Bin Wang, RuiXue Zhang, Li Ping Zhang, Xiao Ping Ma, Chen Wei, ChengRong Song, WenWen Yu, Xiang Wang, Xu Li

**Affiliations:** 1 Center for Translational Medicine, First Affiliated Hospital of Xi’an Jiaotong University, Xi’an 710061, P.R. China; 2 Key Laboratory for Tumor Precision Medicine of Shaanxi Province, First Affiliated Hospital of Xi’an Jiaotong University, Xi’an 710061, P.R. China; 3 Medical Heredity Research Center, Northwest Women’s and Children’s Hospital, Shaanxi, Xi’an 710003, P.R. China; 4 Department of Laboratory Medicine, Central Hospital of Panyu District, Guangzhou, Guangdong 511400, P.R. China; 5 Molecular Diagnosis Department, Guangzhou Lanji Biotechnology Co., Ltd., Guangzhou, Guangdong 510665, P.R. China

**Keywords:** amplification refractory mutation system PCR, gene mutation, next-generation sequencing, phenylalanine hydroxylase, phenylketonuria

## Abstract

To develop a screening kit for detecting mutation hotspots of the phenylalanine hydroxylase (PAH) gene. Thirteen exons of the PAH gene were sequenced in 84 cases with phenylketonuria (PKU) diagnosed during neonatal genetic and metabolic disease screening in Shaanxi province, and their mutations were analyzed. We designed and developed a screening kit to detect nine mutation sites covering more than 50% of the PAH mutations found in Shaanxi province (c.728G>A, c.1197A>T, c.331C>T, c.1068C>A, c.611A>G, c.1238G>C, c.721C>T, c.442-1G>A, and c.158G>A) by using amplification refractory mutation system-polymerase chain reaction (ARMS-PCR) combined with fluorescent probe technology. Peripheral blood and dried blood samples from PKU families were used for clinical verification of the newly developed kit. PAH gene mutations were detected in 84 children diagnosed with PKU. A total of 159 mutant alleles were identified, consisting of 100 missense mutations, 28 shear mutations, 24 nonsense mutations, and 7 deletion mutations. Exon 7 had the highest mutation frequency (32.08%). Among them, the mutation frequency of p.R243Q was the highest, accounting for 20.13% of all mutations, followed by p.R111X, IVS4-1G>A, EX6-96A>G, and p.R413P; these five loci accounted for 47.17% (75/159) of all mutations. In addition, we identified three previously unreported PAH gene mutations (p.C334X, p.G46D, and p.G256D). Fifteen mutation sites were identified in the 47 PAH carriers identified by next-generation sequencing (NGS), which were verified by the newly developed kit, with an agreement rate of 100%. This newly developed kit based on ARMS-PCR combined with fluorescent probe technology can be used to detect common PAH gene mutations.

## Introduction

Phenylketonuria (PKU) is an autosomal recessive metabolic disease caused by mutations in the gene encoding phenylalanine hydroxylase (PAH) [1]. At present, the early diagnosis and intervention of PKU through neonatal disease screening can avoid the damage to the nervous system and other tissues caused by PAH mutations [2]. At the same time, prenatal diagnosis can reduce the chance of giving birth to children with PKU in high-risk families. From the perspective of the three-level prevention system, the most effective way to prevent PKU is to identify carriers before pregnancy, so as to take effective measures to avoid the birth of PKU children [3]. However, due to the variety and heterogeneity of PAH gene mutations, a lot of basic and clinical research is needed to avoid the birth of children with PKU.

As the incidence of PKU and the spectrum of PAH gene mutations in Shaanxi province, China are not clear, it is necessary to combine PKU prevention strategies with the screening and diagnosis of PAH gene mutations, so as to form an effective three-level prevention model and improve the efficiency of PKU prevention. For gene diagnosis of PKU, the most commonly used detection technologies at present mainly include DNA sequencing, multiplex ligation-dependent probe amplification (MLPA), short tandem repeat (STR) linkage analysis etc. However, there are problems associated with the use of these approaches, such as complex operation, high cost, high requirements for laboratory personnel, and not being conducive to popularization [4,5]. Therefore, the purpose of the present study was to develop amplification refractory mutation system-polymerase chain reaction (ARMS-PCR) combined with fluorescent probe technology to detect the mutation hotspots of the PAH gene, in order to develop a PAH gene screening kit that is convenient, fast, cheap, accurate, and suitable for all levels of screening agencies, thereby providing technical support for the ‘one-level prevention’ of PKU [3].

## Materials and methods

### Screening of PAH gene mutations

#### Research material

Data of newborn PKU screening in Shaanxi province, China, were acquired from the ‘National Newborn Disease Screening Information Direct Reporting System,’ and data were taken from 11 newborn disease screening reports in Shaanxi province from 2010 to 2018. Mutations in the PAH gene were present in 84 cases of PKU diagnosed by Shaanxi Neonatal Screening Center from 2010 to 2018. The patient flow as showed in Supplementary Material S1.

#### Sample collection

According to the requirements of the ‘Technical Specifications for Blood Collection for Neonatal Disease Screening’ issued by the Chinese Ministry of Health [6], blood was collected between 72 h after birth and the first full breast feeding. Peripheral blood (0.5 ml) was collected from the inner (or outer) edge of the heel of the newborn. The first drop of blood was discarded and another drop was applied to osmotic filter paper (903™) for neonatal disease screening (diameter of the blood spot on both sides of the paper ≥ 8 mm). The filters were then air-dried, placed in a sealed bag, and stored at −80°C. The present study was carried out after informed consent was obtained from all subjects and was approved by the Ethics Committee of First Affiliated Hospital of Xi’an Jiaotong University.

#### Polymerase chain reaction amplification

The sequences of primers covering the 13 exons and flanking introns of the PAH gene are shown in Supplementary Material S2. PCR was performed in a total volume of 25 µl, including 1 U Taq Plus DNA polymerase, 1× Taq Plus PCR buffer, 2 pmol/l upstream and downstream primers, 2.5 mmol/l dNTPs, and 50 ng DNA template. The following cycling conditions were used for PCR: 94°C pre-denaturation for 15 min; 11 cycles of 94°C for 45 s, 62°C for 45 s (0.5°C drop per cycle), and 72°C for 1 min; 24 cycles of 94°C for 45 s, 57°C for 45 s, and 72°C for 1 min; and a final step of 72°C for 10 min. The amplified products were detected by 1% agarose gel electrophoresis.

#### Sequencing of PCR products

PCR products were sent to Biotechnology (Shanghai) Co., Ltd. for sequencing. Compared with the PAH genomic DNA sequence (GI: 209364518) in GenBank, the naming of mutations followed the principles of the PAH database (http://www.pahdb.mcgill.ca/). Novel mutations were identified by referring to the Human Gene Mutation Database (http://www.hgmd.org) and related literature. A mutation was considered to be new after screening the exons of 50 unrelated individuals to exclude it as a polymorphic site.

#### Data analysis

Estimated annual percentage change was used to evaluate the trend of PKU screening and morbidity index. A *t* test for two independent samples was used to compare the differences of the neonatal disease screening rate, recall review rate, and PKU morbidity between Shaanxi Neonatal Disease Screening Center and the whole province. *P*<0.05 was considered to indicate statistical significance.

### Development of the ARMS-PCR combined with fluorescent probe technology kit

#### Research material for the single-tube monochromatic fluorescence method

Samples from 218 members of 72 PKU families confirmed in the Pediatrics and Medical Genetics Center of Northwest Women’s and Children’s Hospital, Shaanxi, Xi’an from January 2010 to January 2015 were collected. Samples with incomplete clinical information, unqualified samples, and those in which the mutation site was not within the scope of this test as determined by next-generation sequencing (NGS) were excluded. As a result, 180 members of 58 PKU families were finally collected for the determination of the reaction conditions and performance verification of the kit. All subjects signed an informed consent form. Dried blood samples on filter paper were prepared and stored at −80°C.

#### Research material for the single tube two-color fluorescence method

The positive samples consisted of 126 peripheral blood samples from 42 PKU families that had mutations within the detection sites covered by the kit, which were diagnosed clinically and identified by NGS in the Genetics Center of Northwest Women’s and Children’s Hospital from January 2010 to December 2018. The control samples were randomly selected from a healthy population in the same period, and a total of 50 samples were confirmed as wildtype by NGS. All subjects signed an informed consent form. Dried blood samples on filter paper were prepared and stored at −80°C.

#### Genomic DNA extraction

Genomic DNA was extracted from whole blood according to the instructions of the TIANamp Blood DNA Kit (DP348; TIANGEN). Genomic DNA was extracted from dried blood spots according to the instructions of the TIANamp Genomic DNA Kit (DP334-03; TIANGEN).

#### Design and synthesis of primers and probes

For the single-tube monochromatic fluorescence method, according to the technical principle of ARMS-PCR, two specific upstream primers, one common probe, and one common downstream primer were designed using Primer 5.0 software. The probe was labeled with a 6-carboxyfluorescein (6-FAM) fluorescent dye at the 5′-end and with a black hole quencher 1 (BHQ1) fluorescence quenching group at the 3′-end. The primer probe sequence is shown in Supplementary Material S3. For the single-tube two-color fluorescence method, in which one tube was used to detect the mutation of two sites simultaneously, the detection probe of one site was labeled with a 6-FAM fluorescent dye at the 5′-end, the detection probe of the other site was labeled with a VIC or 5-hexachloro-fluorescein (HEX) fluorescent dye, and the 3′-end was labeled with a BHQ1 fluorescence quenching group; the gene sequences are shown in Supplement 4.

#### Establishment of the ARMS-PCR system (Supplementary Material S5)

#### Components of the ARMS-PCR combined with fluorescent probe technology kit (Supplementary Material S6)

#### Interpretation of the results

For the single-color fluorescence method, if the Δ*C*_T_ value was greater than 5, the sample was considered to be homozygous (the mutation amplification curve is in front, which is homozygous; the wildtype amplification curve is in front, which is wildtype), and if the Δ*C*_T_ value was less than 5, the sample was considered to be heterozygous. For the two-color fluorescence method, according to the probe-labeled fluorescence signal, the mutation sites could be identified, but homozygous and heterozygous mutations could not be distinguished.

## Results

### Overview of PKU screening and diagnosis in Shaanxi province from 2010 to 2018

As shown in Table 1, in 2010–2018, 3252675 newborns were screened in Shaanxi province, with an average screening rate of 87.66% (3252675/3710552); 14202 were positive in the primary screening, with an average positive rate of 0.44%; 569 children were diagnosed with PKU, with an average incidence of 1.5/10000.

**Table 1:** Newborn disease screening and PKU diagnosis in Shaanxi province from 2010 to 2018

Year	Number of live births	Number screened	Screening rate (%)	Positive cases in initial screening	Initial screening positive rate (%)	Confirmed PKU cases	Incidence of PKU (1/10000)
2010	253006	146794	58.00	574	0.39	72	2.8
2011	259840	154018	59.27	604	0.39	76	2.9
2012	382384	308182	80.59	1521	0.49	68	1.8
2013	466226	403888	86.63	1708	0.42	59	1.3
2014	463605	443455	95.65	2062	0.47	87	1.9
2015	443341	426563	96.22	1777	0.42	60	1.4
2016	482866	446435	92.46	2351	0.53	81	1.7
2017	514259	496809	96.61	2193	0.44	33	0.6
2018	445025	426531	95.84	1412	0.33	33	0.7
Total	3710552	3252675	87.66	14202	0.44	569	1.5

### Analysis of PAH gene mutations of 84 PKU patients in Shaanxi province

**Figure 1:**
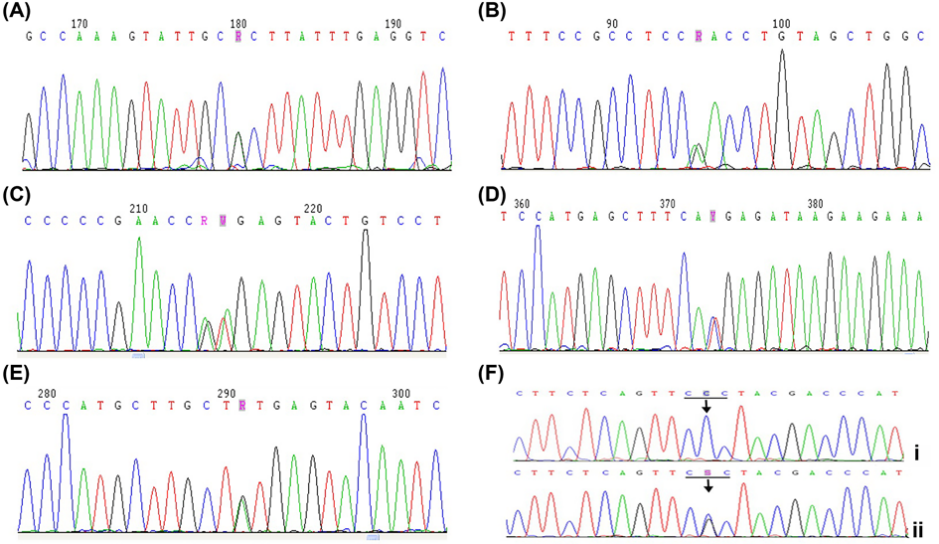
PAH gene mutation sequences (**A**) Sequence map of the p.R53H (c.158G>A) PAH gene mutation. (**B**) Sequence map of the p.R243Q (c.728G>A) PAH gene mutation. (**C**) Sequence map of the IVS7+2T>A (c.842+2T>A) PAH gene mutation. (**D**) Sequence map of the p.R111X (c.331C>T) PAH gene mutation. (**E**) Sequence map of the p.Y204C (c.611A>G) PAH gene mutation. (**F**) Sequence map of the p.R413P (c.1238G>C) PAH gene mutation. I: p.R413P homozygous mutation; II: p.R413P heterozygous mutation (arrow indicates the mutation site; horizontal line shows the codon).

Mutations were detected in 84 children; 5 children had 3 mutations, 65 children had 2 mutations (including 4 homozygotes), and 14 children had 1 mutation. The sequencing results of 6 mutations are shown in Figure 1.

### Analysis of PAH gene mutations in 84 PKU patients

There were 51 kinds of 159 mutated PAH gene alleles in 84 PKU children, consisting of 100 missense mutations, 28 shear mutations, 24 nonsense mutations, and 7 deletion mutations. The mutation detection rate was 94.64% (159/168). The mutation frequency was highest in exon 7, 32.08% (51/159), followed by exons 3 and 12, 13.84% (22/159) and 11.95% (19/159), respectively. Among them, p.R243Q was the most commonly observed mutation, accounting for 20.13% (32/159) of all mutations, followed by p.R111X (8.18%), IVS4-1G>A (6.92%), EX6-96A>G (6.29%), and p.R413P (5.66%), while the other mutations were sporadic (Table 2).

**Table 2:** PAH gene mutations in children with PKU in Shaanxi province, China

Amino acid alteration	Base alteration	Mutation type	Area	Number of alleles	Mutation frequency
p.G46D	c.137G>A	Missense mutation	E2	1	0.62%
p.A47E	c.140C>A	Missense mutation	E2	1	0.62%
p.R53H	c.158G>A	Missense mutation	E2	4	2.51%
p.Ile65Ser	c.194T>G	Missense mutation	E3	2	1.25%
p.Ser70del	c.208-210delTCT	Deletion mutation	E3	6	3.77%
p.Ile95del	c.284-286delTCA	Deletion mutation	E3	1	0.62%
p.R111X	c.331C>T	Nonsense mutation	E3	13	8.18%
p.P147L	c.440C>T	Missense mutation	E4	4	2.51%
IVS4-1G>A	c.442-1G>A	Splice mutation	I4	11	6.92%
p.F161S	c.482T>C	Missense mutation	E5	1	0.62%
p.Y166X	c.498C>A	Nonsense mutation	E5	3	1.88%
p.H170Q	c.510T>A	Missense mutation	E6	1	0.62%
p.G188D	c.562G>A	Missense mutation	E6	2	1.25%
EX6-96A>G	c.611A>G	Splice mutation	E6	10	6.29%
p.V230I	c.688G>A	Missense mutation	E6	1	0.62%
p.V230A	c.689T>C	Missense mutation	E6	1	0.62%
p.R241C	c.721C>T	Missense mutation	E7	4	2.51%
p.R241H	c.722G>A	Missense mutation	E7	2	1.25%
p.L242F	c.724C>T	Missense mutation	E7	1	0.62%
p.R243Q	c.728G>A	Missense mutation	E7	32	20.13%
p.G247R	c.739G>C	Missense mutation	E7	1	0.62%
p.G247V	c.740G>T	Missense mutation	E7	2	1.25%
p.L255S	c.764T>C	Missense mutation	E7	2	1.25%
p.G256D	c.767G>A	Missense mutation	E7	1	0.62%
p.A259T	c.775G>A	Missense mutation	E7	1	0.62%
p.M276K	c.827T>A	Missense mutation	E7	1	0.62%
p.T278I	c.833C>T	Missense mutation	E7	3	1.88%
p.E280K	c.838G>A	Missense mutation	E7	1	0.62%
IVS7+1G>A	c.842+1G>A	Splice mutation	I7	1	0.62%
IVS7+2T>A	c.842+2T>A	Splice mutation	I7	4	2.51%
p.R97C	c.889C>T	Missense mutation	E8	1	0.62%
p.S303P	c.907T>C	Missense mutation	E8	1	0.62%

Amino acid alteration	Basic group alteration	Mutation type	Area	Number of alleles	Mutation frequency
p.A309D	c.926C>A	Missense mutation	E9	1	0.62%
p.I324N	c.971T>A	Missense mutation	E10	1	0.62%
p.W326X	c.977G>A	Nonsense mutation	E10	1	0.62%
p.C334X	c.1002C>A	Nonsense mutation	E10	1	0.62%
p.A342T	c.1024G>A	Missense mutation	E10	2	1.25%
p.A345T	c.1033G>A	Missense mutation	E10	1	0.62%
p.Y356X	c.1068C>A	Nonsense mutation	E11	6	3.77%
p.Y387D	c.1159T>G	Missense mutation	E11	1	0.62%
P.V399V	c.1197A>T	Missense mutation	E11	2	1.25%
p.R400T	c.1199G>A	Missense mutation	E11	4	2.51%
IVS11+1G>C	c.1199+1G>C	Splice mutation	I 11	1	0.62%
p.R408W	c.1222C>T	Missense mutation	E12	2	1.25%
p.R408Q	c.1223G>A	Missense mutation	E12	3	1.88%
p.F410S	c.1229T>C	Missense mutation	E12	1	0.62%
p.R413P	c.1238G>C	Missense mutation	E12	9	5.66%
p.T418P	c.1252A>C	Missense mutation	E12	1	0.62%
p.Q419R	c.1256A>G	Missense mutation	E12	1	0.62%
p.A434D	c.1301C>A	Missense mutation	E12	2	1.25%
IVS12+2T>C	c.1315+2 T>C	Splice mutation	I12	1	0.62%
Total				159	100%

### Discovery of new PAH gene mutations

**Figure 2:**
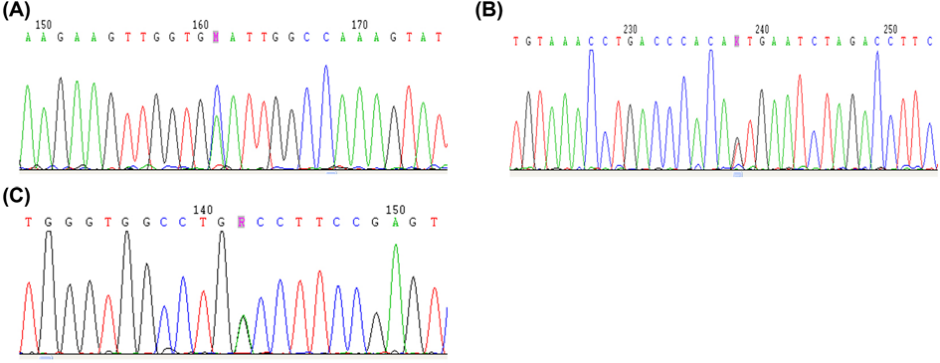
Sequence maps of PAH gene mutations (**A**) Sequence map of the p.A47E (c.140C>A) PAH gene mutation. (**B**) Sequence map of the p.I65S (c.194T>G) PAH gene mutation. (**C**) Sequence map of the p.A259T (c.775G>A) PAH gene mutation.

**Figure 3:**
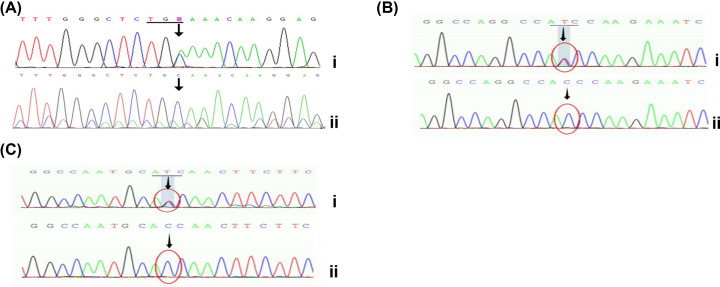
Sequence maps of exons 10, 2, and 7 of the PAH gene (**A**) Sequencing map of exon 10 of the PAH gene; I: p.C334X mutation (c.1002C>A, arrow indicates the mutation site; horizontal line shows the codon); II: normal gene sequence. (**B**) Sequencing map of exon 2 of the PAH gene; I: p.G46D mutation (c.137G>A, arrow indicates the mutation site; horizontal line shows the codon); II: normal gene sequence. (**C**) Sequence map of exon 7 of the PAH gene; I: p.G256D mutation (c.767G>A, arrow indicates the mutation site; horizontal line shows the codon); II: normal gene sequence.

Three missense mutations of the PAH gene (p.A47E, p.I65S, and p.A259T; Figure 2), were found for the first time in the Chinese population. By comparing information held in the PAH database, Human Gene Mutation Database, and ClinVar (https://www.ncbi.nlm.nih.gov/clinvar/), we identified three previously unreported PAH gene mutations (p.C334X, p.G46D, and p.G256D; Figure 3). Among them, p.C334X (c.1002C>A) is a TGC mutation of the Cys codon on exon 10, resulting in the early termination of protein translation, leading to the loss of the partial catalytic and C-terminal tetramer regions, thereby generating a protein that is unable to a form a tetramer and lacks catalytic activity.

### Verification of the ARMS-PCR combined with fluorescent probe technology system

According to the PAH mutations in Shaanxi province and the mutations of the PAH gene in China, we selected nine mutation sites covering greater than 50% of the mutations of the PAH gene in Shaanxi province: c.728G>A (p.R243Q), c.1197A>T (p.V399V), c.331C>T (p.R111X), c.1068C>A (p.Y356X), c.611A>G (EX6-96A>G), c.1238G>C (p.R413P), c.721C>T (p.R241C), c.442-1G>A (IVS4-1G>A), and c.158G>A (p.R53H). We used ARMS-PCR combined with fluorescent probe technology to design and develop a kit to detect common mutations of the PAH gene. Refer to **Supplementary Material S7** for a comparison between the results of the monochromatic fluorescence method and NGS. After exploring the experimental conditions, optimizing the experimental procedures, and calibrating the temperature control system of the instrument, 117 samples from 40 families were used to verify the performance of the ARMS-PCR combined with fluorescent probe technology system, and the results were compared with those from NGS (Table 3). Taking NGS as the gold standard, the detection sensitivity of ARMS-PCR combined with fluorescent probe technology was 94.17% (97/103). Fifty-three samples without a mutation as determined by NGS were also found to be negative with the newly developed approach, indicating that the detection specificity of the ARMS-PCR combined with fluorescent probe technology system can reach 100%.

**Table 3:** Comparison of PCR detection and sequencing results for 117 individuals from 40 families

Methodological comparison	NGS		
	Positive (mutation sites were detected)	Negative (wildtype)	Total
ARMS-PCR-positive	97	0	97
ARMS-PCR-negative	6	53	59
Total	103	53	156

Six mutation sites were not detected by ARMS-PCR combined with fluorescent probe technology (twice each for c.158G>A, c.331C>T, and c.442-1G>A) which was inconsistent with the results of NGS. Factors, such as experimental operation, instrument stability, and repeated freezing and thawing of the reagents, were excluded as contributing to the lack of detection. As such, we considered that the sensitivity of the detection reagents needs to be improved.

### Verification of the two-color fluorescence method

The results of ARMS-PCR combined with fluorescent probe technology were compared with the results of NGS (**Supplementary Material S8**). We found that when DNA extracted from dried blood spots was used as the template and the concentration was approximately 3 ng/µl, the kit could detect the mutation sites of the positive samples with the two-color fluorescence method, the Δ*C*_T_ value was less than 35, and the wildtype samples were negative. At the same time, the detection results were consistent with the known positive samples. When DNA extracted from whole blood was used as the template and the concentration was 100–200 ng/µl, using the two-color fluorescence method, the kit could detect the mutation sites of each positive sample, the Δ*C*_T_ value was less than 32, and the wildtype samples were negative. At the same time, the detection results of the new approach were consistent with the known positive sample gene mutation sites (**Supplementary Material S9**). The kit was used to analyze whole blood and dried blood spot samples from 126 cases confirmed by sequencing as PKU. After DNA extraction and PCR amplification, ARMS-PCR combined with fluorescent probe technology could accurately detect the positive samples, and the positive mutation sites detected by the kit were exactly the same as those detected by NGS (Table 4).

**Table 4:** Summary of PCR and sequencing results for 126 clinical samples

Detection site	Number of cases	Coincidence rate with sequencing results (%)
728	47	100
611	13	100
442-1	20	100
1238	8	100
1068	5	100
331	8	100
721	2	100
1197	10	100
158	13	100
Total	126	100

## Discussion

The present study analyzed the general situation of neonatal disease screening and PKU incidence in Shaanxi province, China from 2010 to 2018. The screening rate of neonatal diseases increased from 58% in 2010 to 95% in 2018. In the past 9 years, 569 cases of PKU were diagnosed. Through treatment with a low phenylalanine diet, the prognosis was good, and the ultimate goal of early detection and treatment was achieved. Based on this, the incidence of PKU in our province was approximately 1.5/10000, which is basically consistent with the literature [7]. but higher than the national average (0.9/10000). Therefore, it is of great importance to carry out a three-level prevention strategy for PKU in our province to lower its incidence.

In the present study, 13 exons and their flanking sequences of the PAH gene were studied in 84 children with PKU in Shaanxi province, and the mutation spectrum of the PAH gene in Shaanxi province was revealed for the first time. A total of 51 kinds of 159 mutant alleles were found, and the most common type of mutation was missense, accounting for 62.89% (100/159) of all mutations. Exon 7 was the most widely mutated exon, accounting for 32.08% (51/159) of all cases; p.R243Q had the highest mutation frequency, accounting for 20.13% (32/159) of all mutations. In conclusion, the mutations of the PAH gene in Shaanxi province found in the present study were basically consistent with previous reports from China and abroad: exon 7 is a mutation hotspot, especially the p.R243Q mutation, which should be given more attention [8].

A deficiency of PAH caused by gene mutation is the main cause of PKU [9]. Gene detection is an important method to determine the cause of PKU and a guide for family reproduction. Although PAH gene mutations were detected in 84 children with PKU, only 1 mutation was detected in 14 of them. Considering the genetic characteristics of PKU, generally 2 or more mutations in the gene locus are required to cause disease [10]. Therefore, additional gene detection in these 14 children needs to be performed, and it cannot be used to provide reproductive guidance for their families. Reasons for this situation include the possible deletion or duplication of large segments of the PAH gene, which needs to be detected by MLPA. Due to the limitation of detection methods, the present study did not use MLPA to analyze these 14 children, which is one of the shortcomings of the present study. We plan to complete this analysis in a future study. In addition, there may be mutations in other areas of the PAH gene in addition to those analyzed in the present study, and the limitations of the detection methods cannot be determined at the present time. Thus, further research is needed.

As stated earlier, the most common molecular basis of PKU is mutation of the PAH gene, so molecular diagnosis using the PAH gene is key to reducing the number of children born with this disease. According to research in China and abroad, 1101 mutations have been identified in the PAH gene, with obvious heterogeneity. There are significant differences in the location and distribution of PAH loci among different races and regions [11,12]. We detected PAH gene mutations in 84 children with PKU diagnosed in our center and created a gene mutation map of PAH in Shaanxi province. In the present study, ARMS-PCR combined with fluorescent probe technology was used to detect the mutation sites. We designed detection methods using monochromatic and bichromatic fluorescence. Based on the results of the present study, together with the conclusions of studies in other regions in China, we selected the mutation hotspots of the PAH gene with a high incidence in China and developed a detection kit targeting these mutation sites suitable for clinical use. We selected nine sites with a high mutation rate, which contained more than 50% of the mutations identified in Shaanxi province. The identification of 126 positive samples showed that the consistency between the two-color fluorescence amplification technology and NGS was 100%.

On the basis of the common mutations of the PAH gene in Shaanxi province, through many tests, optimization of the conditions, and performance verification, we have developed a PAH gene mutation screening kit, which lays the foundation for a clinical pathway of the three-level prevention system of ‘premarital, pre-pregnancy, pregnancy healthcare/prenatal diagnosis and neonatal genetic metabolic disease screening/and treatment.’ However, the complexity of PAH gene mutations means that the detection of all PAH gene mutation sites cannot be realized by the current ARMS-PCR combined with fluorescent probe technology detection kit. In the present study, the results showed that the kit designed could cover the common mutation sites of PAH and the specificity of detection was 100%. While it is not possible to screen for mutations in multiple diseases at once, compared with second-generation sequencing, it is important for screening carriers of a single genetic disease with a high incidence in a region, the utility model has the advantages of low cost, simple operation, easy interpretation of the results, high specificity, and easy popularization.

With the deepening of our understanding of the mutation spectrum, we will analyze the common mutation sites of the PAH gene again, with an aim to optimize the mutation detection sites and further improve the mutation detection rate.

## Conclusion

This newly developed kit based on ARMS-PCR combined with fluorescent probe technology can be used to detect common PAH gene mutations.

## Supplementary Material

Supplementary Materials S1-S9

## Data Availability

The raw data supporting the conclusions of this article will be made available by the authors upon request.

## Abbreviations

6-FAM: 6-carboxyfluorescein
ARMS-PCR: amplification refractory mutation system-polymerase chain reaction
BHQ1: black hole quencher 1
MLPA: multiplex ligation-dependent probe amplification
NGS: next-generation sequencing
PAH: phenylalanine hydroxylase
PCR: polymerase chain reaction
PKU: phenylketonuria
STR: short tandem repeat
