# Supplementary material for: Development of a mutation hotspot detection kit for the phenylalanine hydroxylase gene by ARMS-PCR combined with fluorescent probe technology
Source: Biosci Rep. 2021 Feb 19;41(2):BSR20201660. doi: 10.1042/BSR20201660 (PMC7897920; doi:10.1042/BSR20201660)
Supplement: Supplementary Materials S1-S9 [file BSR-2020-1660_supp.pdf]

Supply1. The patient workflow

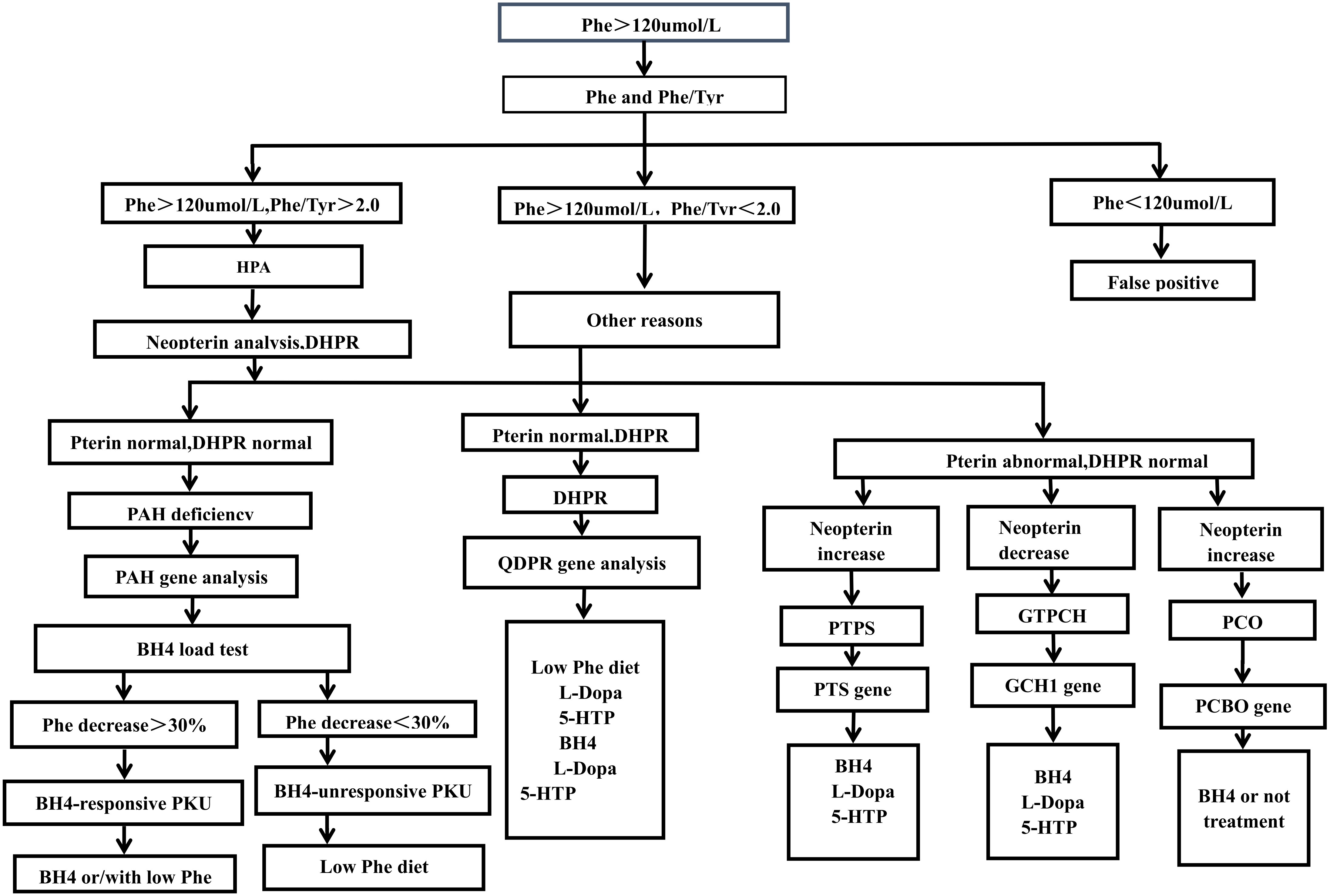

Supply 2 PCR primer sequences of 13 exons of PAH genes

exon	primer（5’-3’）	Product length（bp）
1	F:CCTCCTGCGTCAGGACAAC<br>R:TTCGTTGGAAGCTGATGAGAA	340
2	F:TTGCTTTGTCCATGGAGGTTT<br>R:ACAGGATCTGGAACAGGCAGA	251
3	F:TGTGAACTAACTGCCCCACCT<br>R:TTGCTGTTATTTTGCGGAAGC	495
4	F:GGGATCCCCACTTCTGATCTC<br>R:AACAACTCTGCCAACTGCAGG	505

Supply 2（continued）

exon	primer（5’-3’）	Product length（bp）
5	F:CCCCCATTCAAAGCATTCATA<br>R:CATTCAAGATTTCAGGCCAGG	543
6	F:CCCTTTCATGTGGGAAATCAA<br>R:GTGCTTGTAGGAATGCATGCA	481
7	F:ATGTCCCTGGGCAGTTATGTG<br>R:TGAGAACAGGAACAAGTGGCA	512
8	F:GGGAGCATGTCCACAGGAATA<br>R:TATGATCCCACCTGAAATGGG	470
9	F:GGCCACCCATCACCTTTTTAT<br>R:GTAGCCCTTGAAAACCCTTGG	387
10	F:TCCCTTCATCCAGTCAAGGTG<br>R:ATTCCAAGGCTGACCTATGCA	379
11	F:CAGCATTTGGGCTGTGATGTA<br>R:CGTTCTCTGTTGGAAGGTTGG	400
12	F:ACCCTGCTCTAGGGAGGTGTC<br>R:CCTCTCCATCCCTTCTACGCT	502
13	F:AGCCCACTTATCCCCTAGTGC<br>R：ATTTGGGACCTGCTTCATTCA	413

PAH：phenylalanine hydroxylase

Supply 3.Monochromatic fluorescent ARMS-PCR primer probe sequence

Mutation site		sequence（5'- -3'）
c.728G>A	WF	GCACTGGTTTCCGCCTCCG
	MF	GCACTGGTTTCCGCCTCCA
	R	GGCAACTGGTAGCTGGAGGA
	P	FAM-TCCAAGCCCATGTATACCCCCGAAC-BHQ1
c.1197A>T	WF	TTAATGATGCCAAGGAGAAAGTA
	MF	TTAATGATGCCAAGGAGAAAGTT
	R	ATGAGTGGCACCAGTCAGGAG
	P	FAM -TGACAAAGGTGAGCCACTAGCTCTGGG-BHQ1
c.331C>T	WF	CCACTGTCCATGAGCTTTCAC
	MF	CCACTGTCCATGAGCTTTCAT
	R	ACAGTGTGGAGTTACTTATGTTGCAAA
	P	FAM -ACACAGGTAAGAATTAGAG-BHQ1
c.1068C>A	WF	TCACTTGGGGCCTACAGTAC
	MF	TCACTTGGGGCCTACAGTAA
	R	ACTCCGTGACAGTGTAATTTTGGA
	P	FAM -CCCCTGGAGCTGGAGAAGACAGCC-BHQ1
c.611A>G	WF	GTATAAAACCCATGCTTGCTA
	MF	GTATAAAACCCATGCTTGCTG
	R	CCTCAATCCTCCCCCAACTT
	P	FAM-CACATCAGGGTCAATGGCCCTGC-BHQ1
c.1238G>C	WF	CCTCGGCCCTTCTCAGTTCG
	MF	CCTCGGCCCTTCTCAGTTCC
	R	GGCGATGGTAGGGAAAGACA
	P	FAM -AGGCCACTCGGTTTCTCAGTAATCGAAGA-BHQ1
c.721C>T	WF	ATCCCAGCTTGCACTGGTTTCC
	MF	ATCCCAGCTTGCACTGGTTTCT
	R	GGCAACTGGTAGCTGGAGGA
	P	FAM-TCCAAGCCCATGTATACCCCCGAAC-BHQ1

ARMS-PCR: amplification refractory mutation system-polymerase chain reaction

mutation site		sequence（5'-　　-3'）
c.158G>A	WF	GTGCATTGGCCAAAGTATTGCG
	MF	GTGCATTGGCCAAAGTATTGCA
	R	CAAATTCAAATCTGCCTGTTCCA
	P	FAM-TCTTGGATAATGTCGTAGCAAACTTCCATGTTCT-BHQ1
c.442-1G>A	WF	AATCAGGTGTCTCTTTTCTCCTAG
	MF	AATCAGGTGTCTCTTTTCTCCTAA
	R	GGCAGACTTACTGGCGGTAGTT
	P	FAM -CGGAAGCAGTYTGCTGACATTGCCT-BHQ1
β-actin	F	GCATGGGTCAGAAGGATTCCT
	R	TCGTCCCAGTTGGTGACGAT
	P	FAM-CCTCACCCTGAAGTACCCCATCGAGC-BHQ1

Supply 4. Bichromatic fluorescent ARMS-QPCR primer probe sequence

mutation site		sequence（5'- -3'）
c.728G>A	MF	GCACTGGTTTCCGCCTCCA
	R	GGCAACTGGTAGCTGGAGGA
	P	FAM-TCCAAGCCCATGTATACCCCCGAAC-BHQ1
c.1238G>C	MF	GTATAAAACCCATGCTTGCTG
	R	CCTCAATCCTCCCCCAACTT
	P	VIC-CACATCAGGGTCAATGGCCCTGC-BHQ1
c.331C>T	MF	TTAATGATGCCAAGGAGAAAGTT
	R	ATGAGTGGCACCAGTCAGGAG
	P	FAM -TGACAAAGGTGAGCACTAGCTCTGGG-BHQ1
c.1197A>T	MF	CCTCGGCCCTTCTCAGTTCC
	R	GGCGATGGTAGGGAAAGACA
	P	VIC -AGGCCACTCGGTTTCTTAATCGAAGA-BHQ1
c.611A>G	MF	TCACTTGGGGCCTACAGTAA
	R	ACTCCGTGACAGTGTAATTTTGGA
	P	FAM -CCCCTGGAGCTGGAGAAGACAGCC-BHQ1

（continued）

mutation site		sequence（5'- -3'）
c.1068C>A	MF	CCACTGTCCATGAGCTTTCAT
	R	ACAGTGTGGAGTTACTTATGTTGCAAA
	P	VIC -ACACAGGTAAGAATTAGAG-BHQ1
c.721C>T	MF	ATCCCAGCTTGCACTGGTTTCT
	R	GGCAACTGGTAGCTGGAGGA
	P	FAM-TCCAAGCCCATGTATACCCCCGAAC-BHQ1
c.158G>A	MF	GTGCATTGGCCAAAGTATTGCA
	R	CAAATTCAAATCTGCCTGTTCCA
	P	VIC-TCTTGGATAATGTAGCACTTCCATGTTCT-BHQ1
c.442-1G>A	MF	AATCAGGTGTCTCTTTTCTCCTAA
	R	GGCAGACTTACTGGCGGTAGTT
	P	FAM -CGGAAGCAGTYTGCTGACATTGCCT-BHQ1
gene β-actin	F	GCATGGGTCAGAAGGATTCCT
	R	TCGTCCCAGTTGGTGACGAT
	P	VIC -CCTCACCCTGAAGTACCCCATCGAGC-BHQ1

Note：MF ：Mutant upstream primer； P ：probe；R：Reverse primer；F：Forward primer。

Supply5.

Supply5.1 Monochromatic fluorescence reaction system

composition	The dosage
10×PCR Mix	9.5μl
F/R/P	3μl
DNA	4μl
ddH_2O	33.5μl
The total volume	50μl

Supply5.2 Two-color fluorescence reaction system

composition	The dosage
10×PCR Mix	9.5μl
F1/R1/P1/F2/R2/P2	3μl
Whole blood DNA/dried blood spots DNA	2μl/ 3μl
ddH_2O	35.5μl/ 34.5μl
The total volume	50μl

Supply5.3 Monochromatic fluorescence reaction condition

temperature	time	
95℃	3min	
95℃	15s	Ten cycle
60℃	40s	
95℃	15s	Thirty cycle, this procedure collects fluorescence
60℃	40s	

Supply5.4 Two-color fluorescence reaction condition

temperature	time		
95℃	5min		
95℃	15s	Each cycle goes down by 1 ℃	Nine cycle no fluorescence collection
67℃	15s		
72℃	15s		
95℃	15s	Each cycle goes down by 1 ℃	
67℃	15s		
72℃	15s		
95℃	3min		
95℃	15s	45 cycles (collection fluorescence)	

60.5℃	30s

Supply6.1 components of monochrome fluorescence method kit

composition	Main ingredients
PCR reaction fluid	dNTPs etcs
Enzyme sofe	Taq polymerase
728 Wild Type Detection Fluid	Primers, Probes
728 Mutant Detection Fluid	Primers, Probes
1197 Wild Test Fluid	Primers, Probes
1197 Mutation detection fluid	Primers, Probes
331 Wild Test Liquid	Primers, Probes
331 Mutation detection fluid	Primers, Probes
1068 Wild Test Liquid	Primers, Probes
1068 Mutation Detection Fluid	Primers, Probes
611 Wild Test Liquid	Primers, Probes
611 Mutation detection fluid	Primers, Probes

Supply6.1（continued）

composition	Main ingredients
1238 Wild Test Fluid	Primers, Probes
1238 Mutation Detection Fluid	Primers, Probes
721 Wild Test Liquid	Primers, Probes
721 Mutation detection fluid	Primers, Probes
442-1 Wild Test Liquid	Primers, Probes
442-1 Mutation detection fluid	Primers, Probes
158 Wild Test Liquid	Primers, Probes
158 Mutation detection fluid	Primers, Probes
Positive control（PC）	Plasmid
Blank control（BC）	ddH_2O

Supply6.2 Composition of two-color fluorescence method kits

Ingredients	Tube cover color	Main ingredients
PCR reaction fluid	Yellow	dNTPs etc
Enzyme sofe	Purple	Taq polymerase
Tube 1 detection fluid	Brown	Primers, Probes
Tube 2 detection fluid	Brown	Primers, Probes
Tube 3 Detection Fluid	Brown	Primers, Probes
Tube 4 Detection Fluid	Brown	Primers, Probes

Tube 5 Detection Fluid	Brown	Primers, Probes
Positive control（PC）	Red	Plasmlates
Blank control（BC）	Green	ddH_2O

Supply 7.

A-1

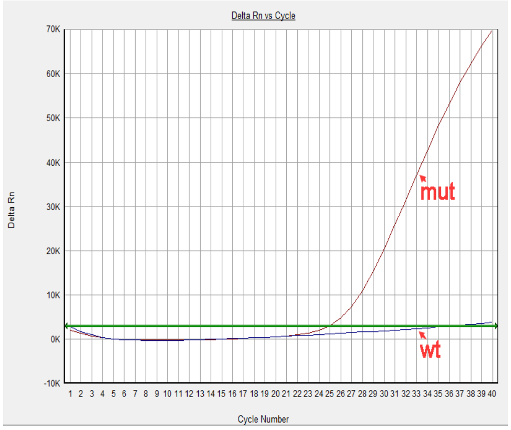

A-2

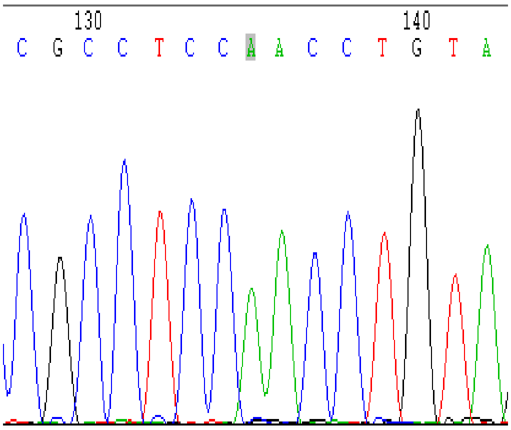

B-1

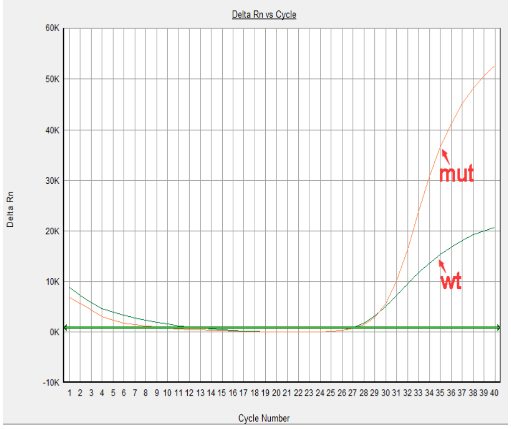

B-2

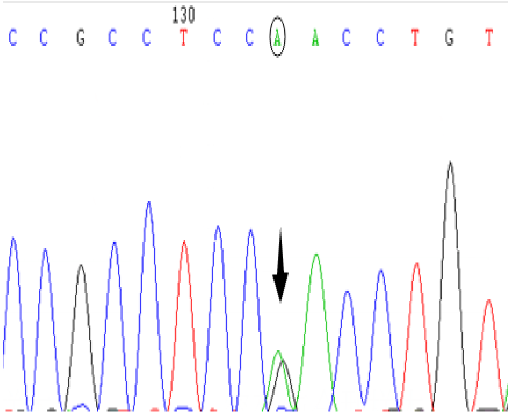

C-1

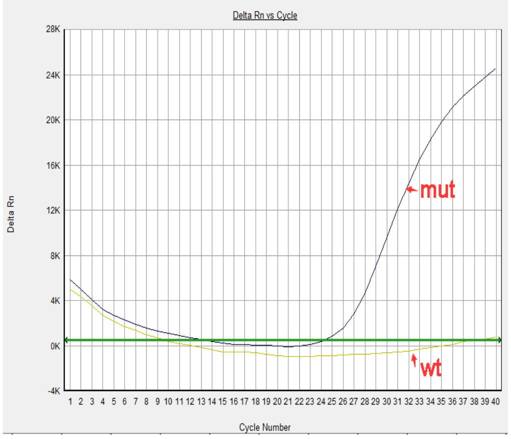

C-2

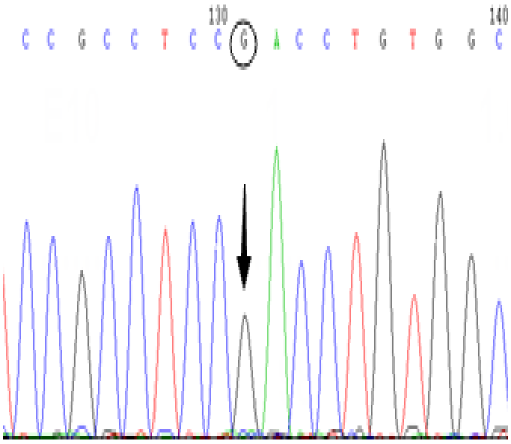

Figure1. ARMS-PCR result and Sequencing result diagram of c.728G>A site

（A-1）:ARMS-PCR results of homozygous mutant samples （A-2）:Sequencing results of homozygous mutant samples;（B-1）:ARMS-PCR results of heterozygous mutant samples （B-2）:Sequencing results of heterozygous mutant samples;

（C-1）:ARMS-PCR results of wild-type samples ; （C-2）:Sequencing results of

wild-type samples.↓ :indicates a mutation site.

A-1

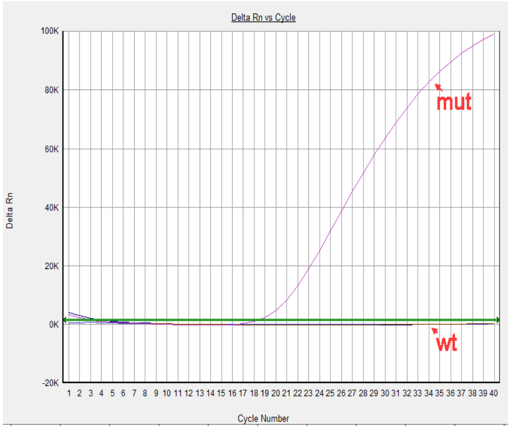

A-2

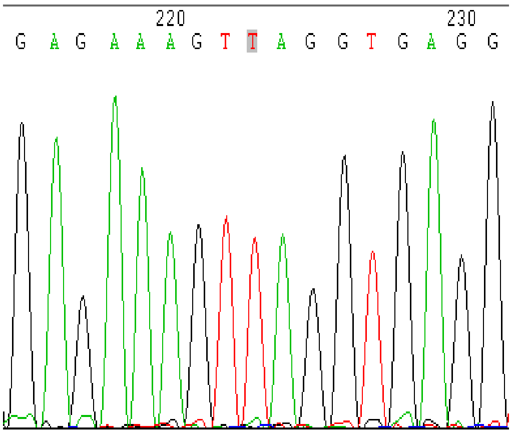

B-1

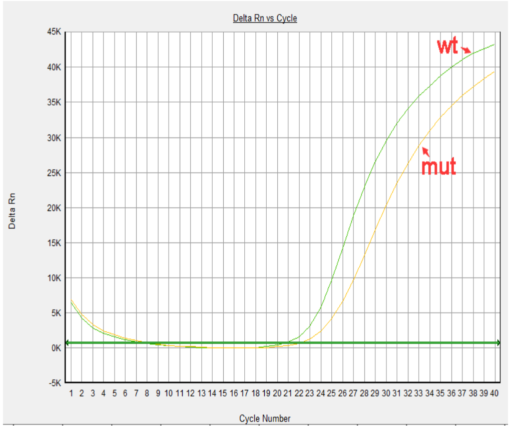

B-2

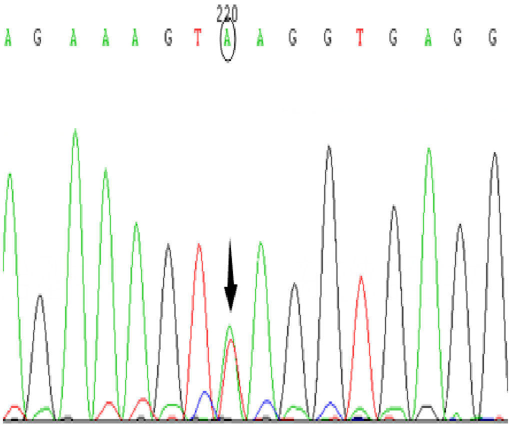

C-1

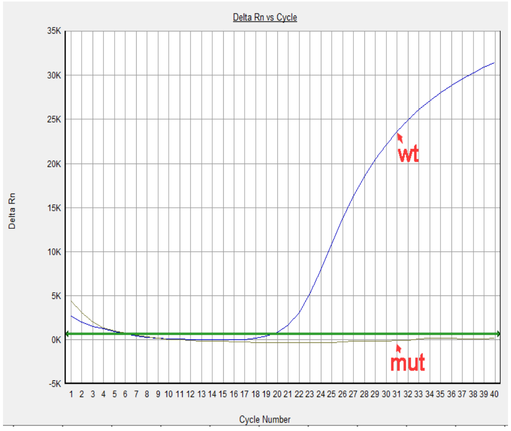

C-2

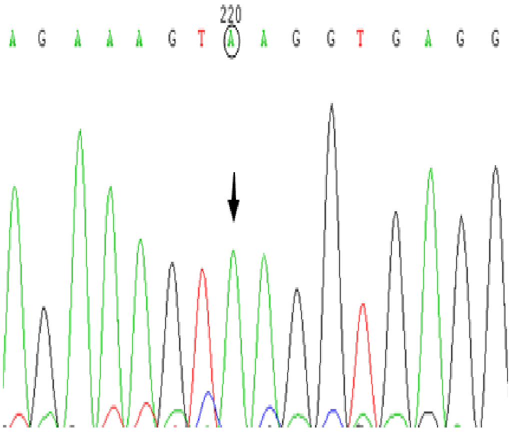

Figure2. ARMS-PCR result and Sequencing result diagram of c.1197A>T site. （A-1）:ARMS-PCR results of homozygous mutant samples; （A-2）:Sequencing results of homozygous mutant samples;

（B-1）:ARMS-PCR results of heterozygous mutant samples; （B-2）:Sequencing results of heterozygous mutant samples; （C-1）:ARMS-PCR results of wild-type samples;（C-2）:Sequencing results of wild-type samples ↓ :indicates a mutation site.

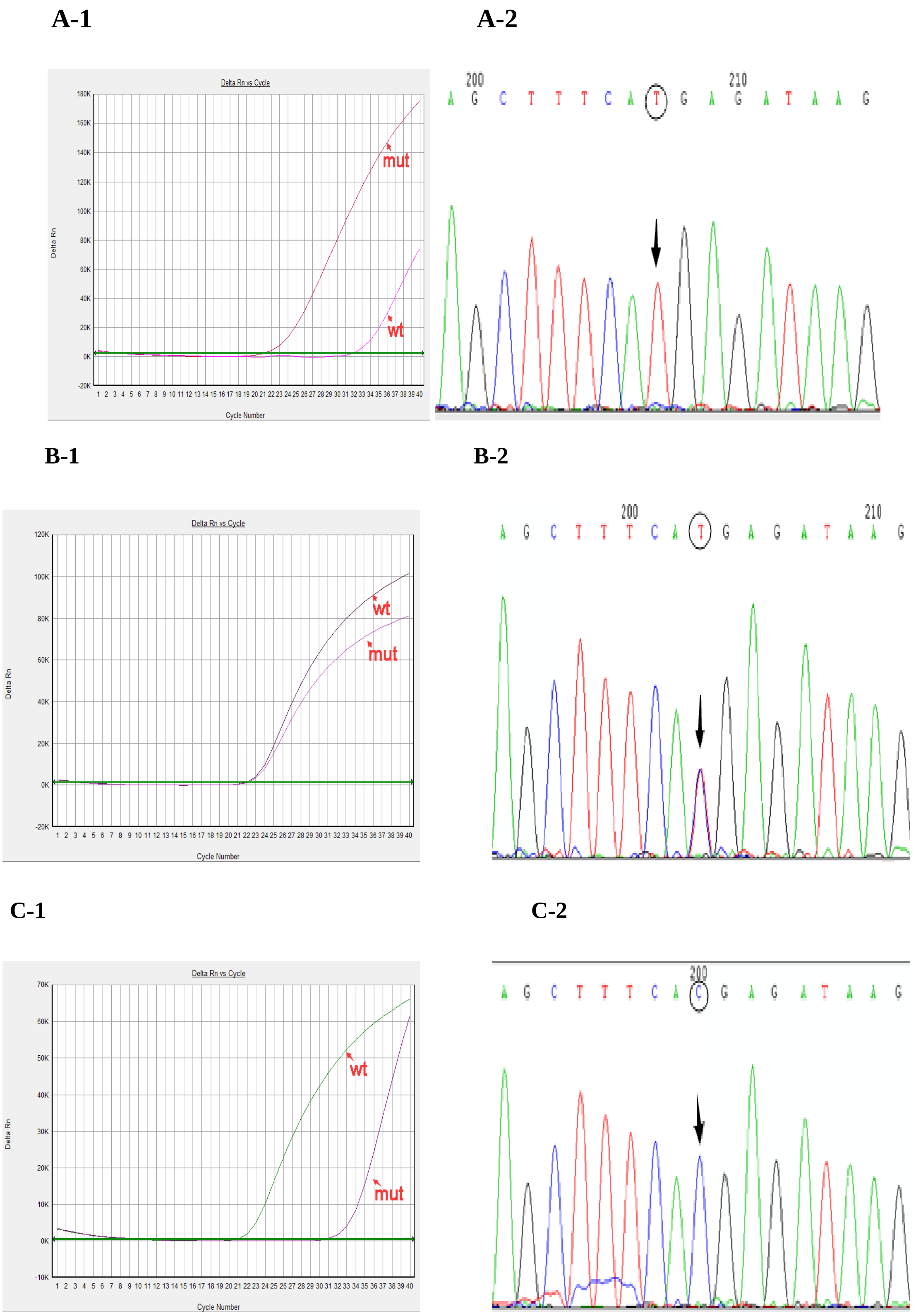

Figure3. ARMS-PCR result and Sequencing result diagram of c.331C>T site

（A-1）:ARMS-PCR results of homozygous mutant samples （B-2）:Sequencing results of homozygous mutant samples（B-1）:ARMS-PCR results of heterozygous mutant samples; （B-2）:Sequencing results of heterozygous mutant samples; （C-1）:ARMS-PCR results of wild-type samples; （C-2）:Sequencing results of wild-type samples ↓:indicates a mutation site.

A-1

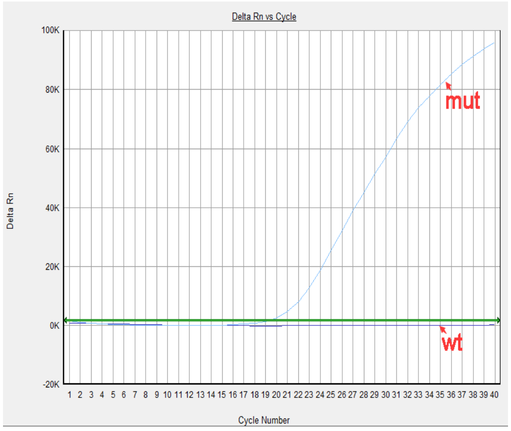

A-2

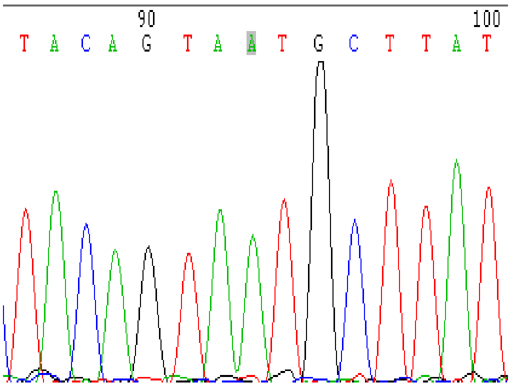

B-1

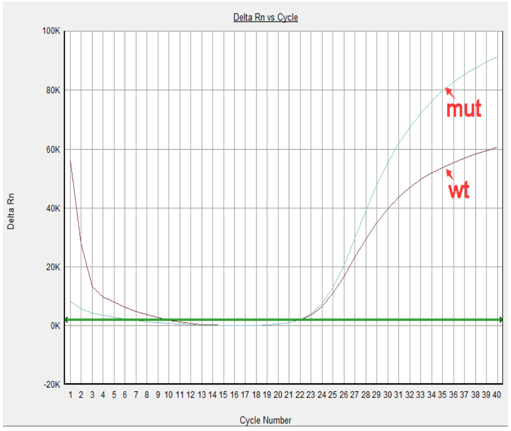

B-2

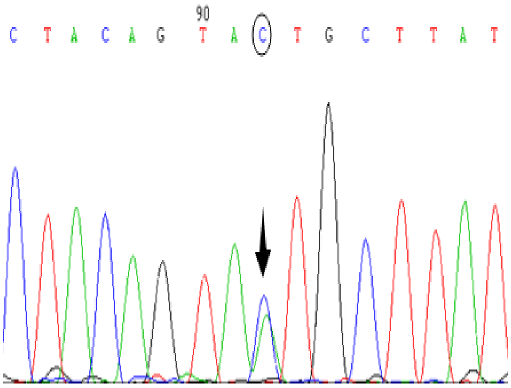

C-1 **C-2**

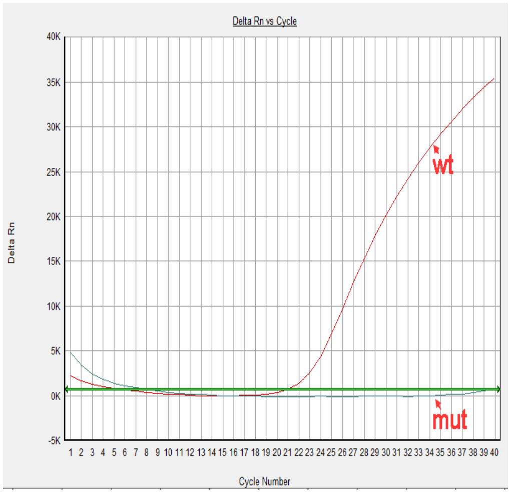

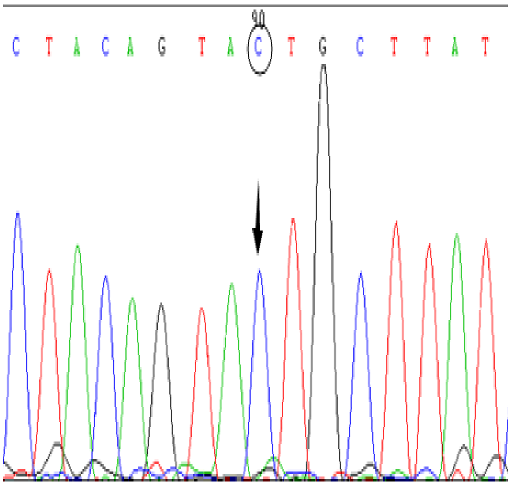

Figure4. ARMS-PCR result and Sequencing result diagram of c.1068C>A site

（A-1）:ARMS-PCR results of homozygous mutant samples. （A-2）:Sequencing results of homozygous mutant samples（B-1）:ARMS-PCR results of heterozygous mutant samples. （B-2）:Sequencing results of heterozygous mutant samples

（C-1）:ARMS-PCR results of wild-type samples.（C-2）:Sequencing results of wild-type samples. ↓ :indicates a mutation site.

A-1 **A-2**

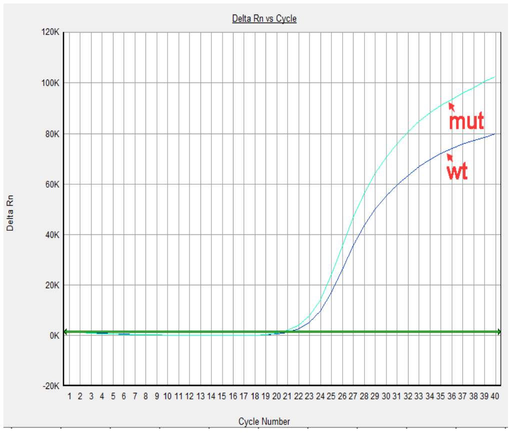

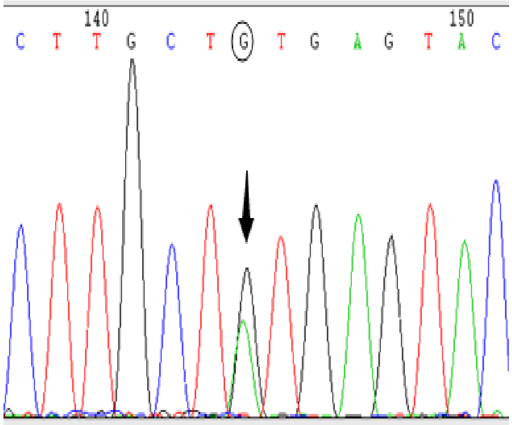

B-1

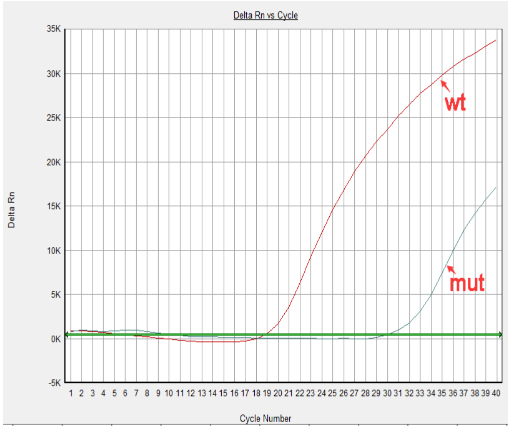

B-2

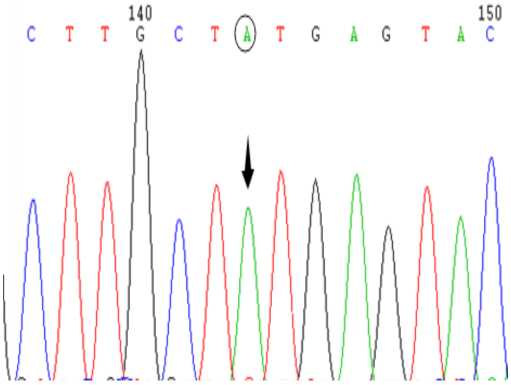

Figure5. ARMS-PCR result and Sequencing result diagram of c.611A>G site

（A-1）:ARMS-PCR results of heterozygous mutant samples. （A-2）:Sequencing results of heterozygous mutant samples.（B-1）:ARMS-PCR results of wild-type samples .（B-2）:Sequencing results of wild-type samples. ↓:indicates a mutation site.

A-1

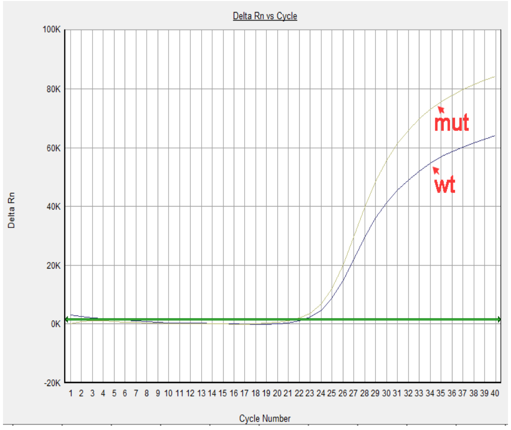

A-2

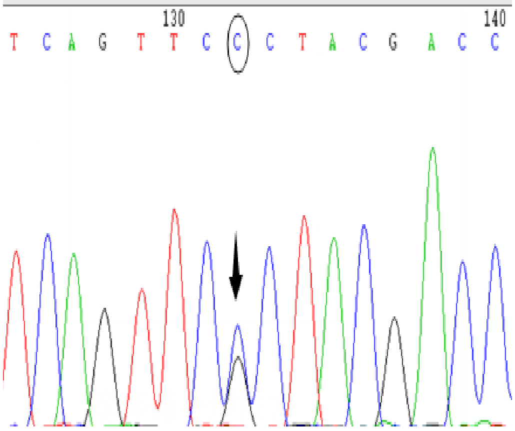

B-1

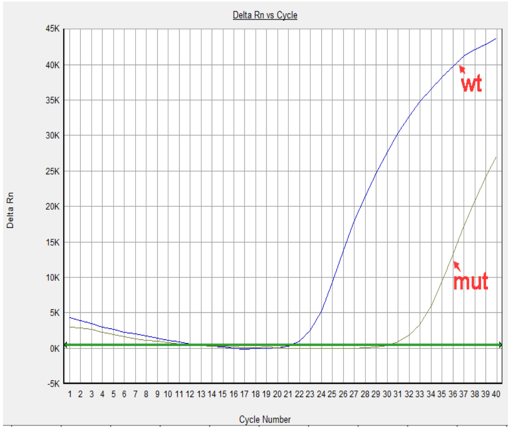

B-2

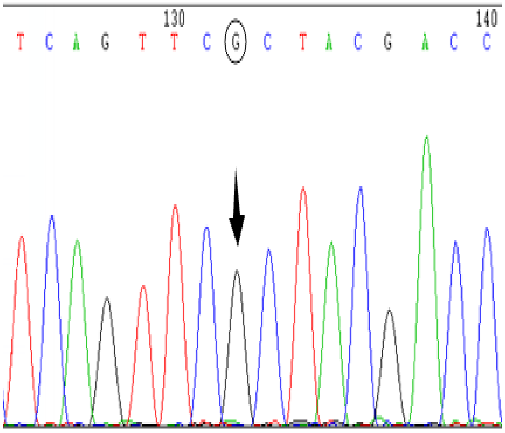

Figure6. ARMS-PCR result and Sequencing result diagram of c.1238G>C.

（A-1）:ARMS-PCR results of heterozygous mutant samples.（A-2）:Sequencing results of heterozygous mutant samples.（B-1）:ARMS-PCR results of wild-type samples.（B-2）:Sequencing results of wild-type samples . ↓:indicates a mutation site.

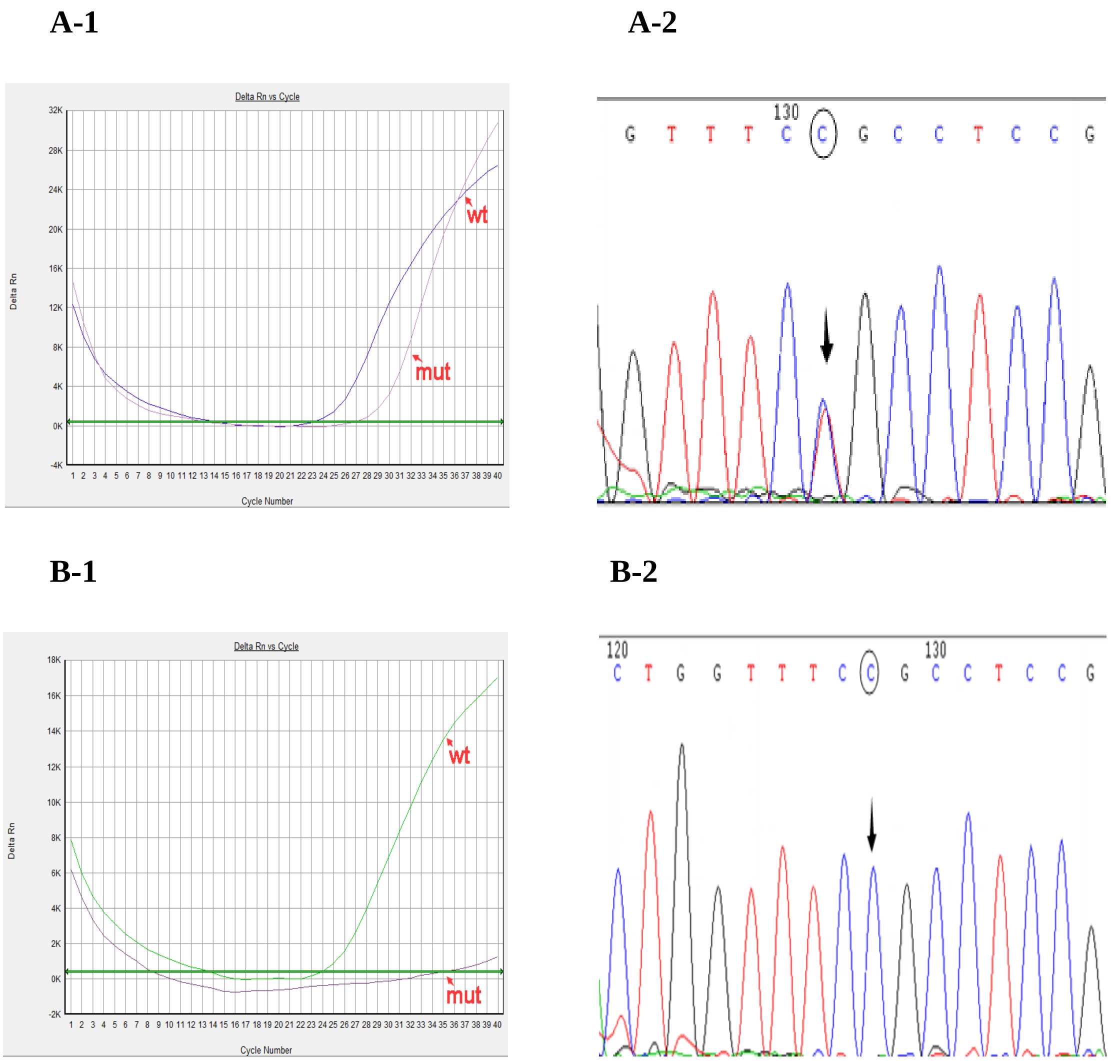

Figure7. ARMS-PCR result and Sequencing result diagram of c.721C>T site.

（A-1）:ARMS-PCR results of heterozygous mutant samples. （A-2）:Sequencing results of heterozygous mutant samples.（B-1）:ARMS-PCR results of wild-type samples. （B-2）:Sequencing results of wild-type samples↓:indicates a mutation site.

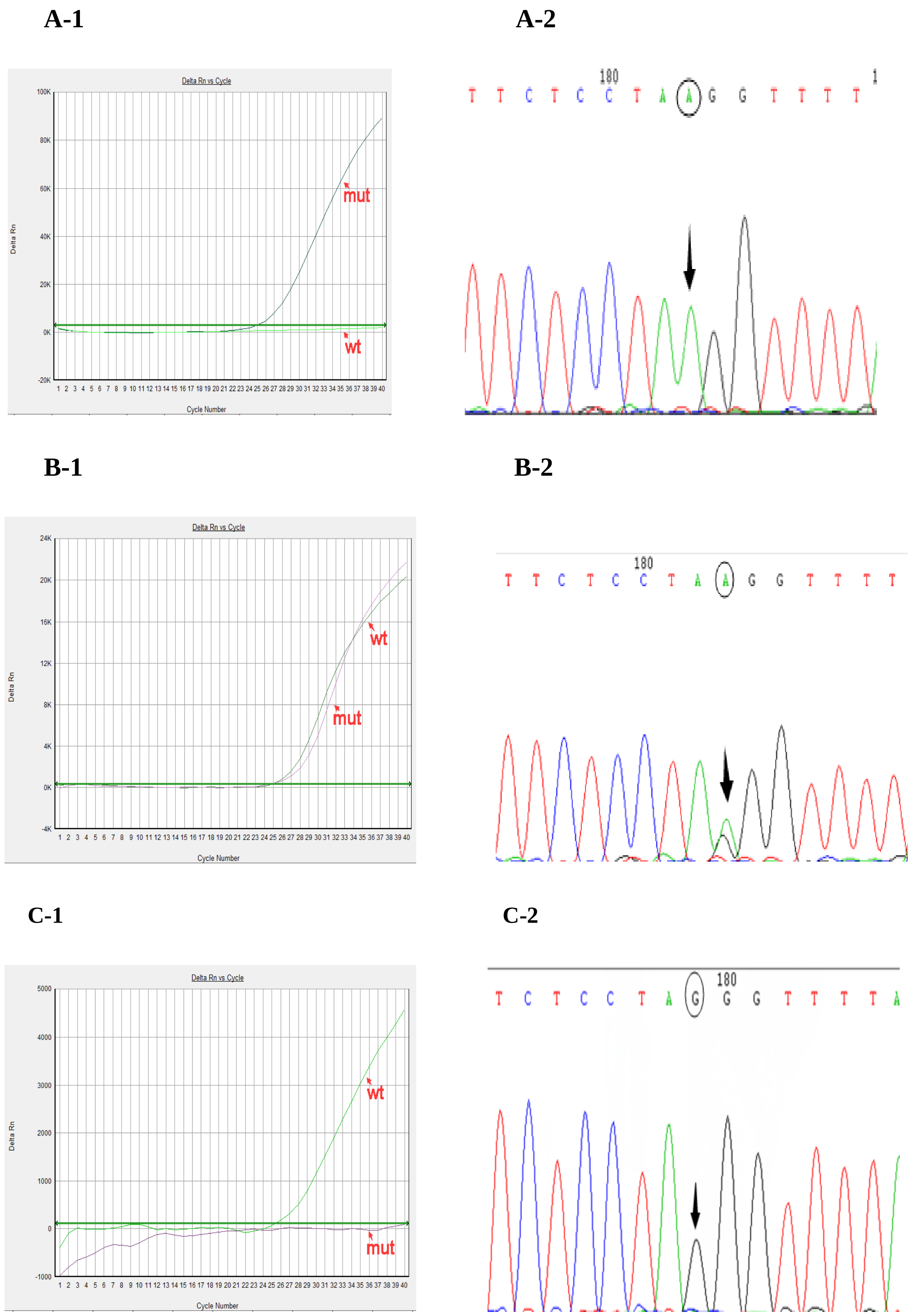

Figure8. ARMS-PCR result and Sequencing result diagram of c.442-1G>A site.

（A-1）:ARMS-PCR results of homozygous mutant samples. （A-2）:Sequencing results of homozygous mutant samples

(B-1) :ARMS-PCR results of heterozygous mutant samples. (B-2) :Sequencing results of heterozygous mutant samples. (C-1) :ARMS-PCR results of wild-type samples. (C-2) :Sequencing results of wild-type samples. ↓ :indicates a mutation site.

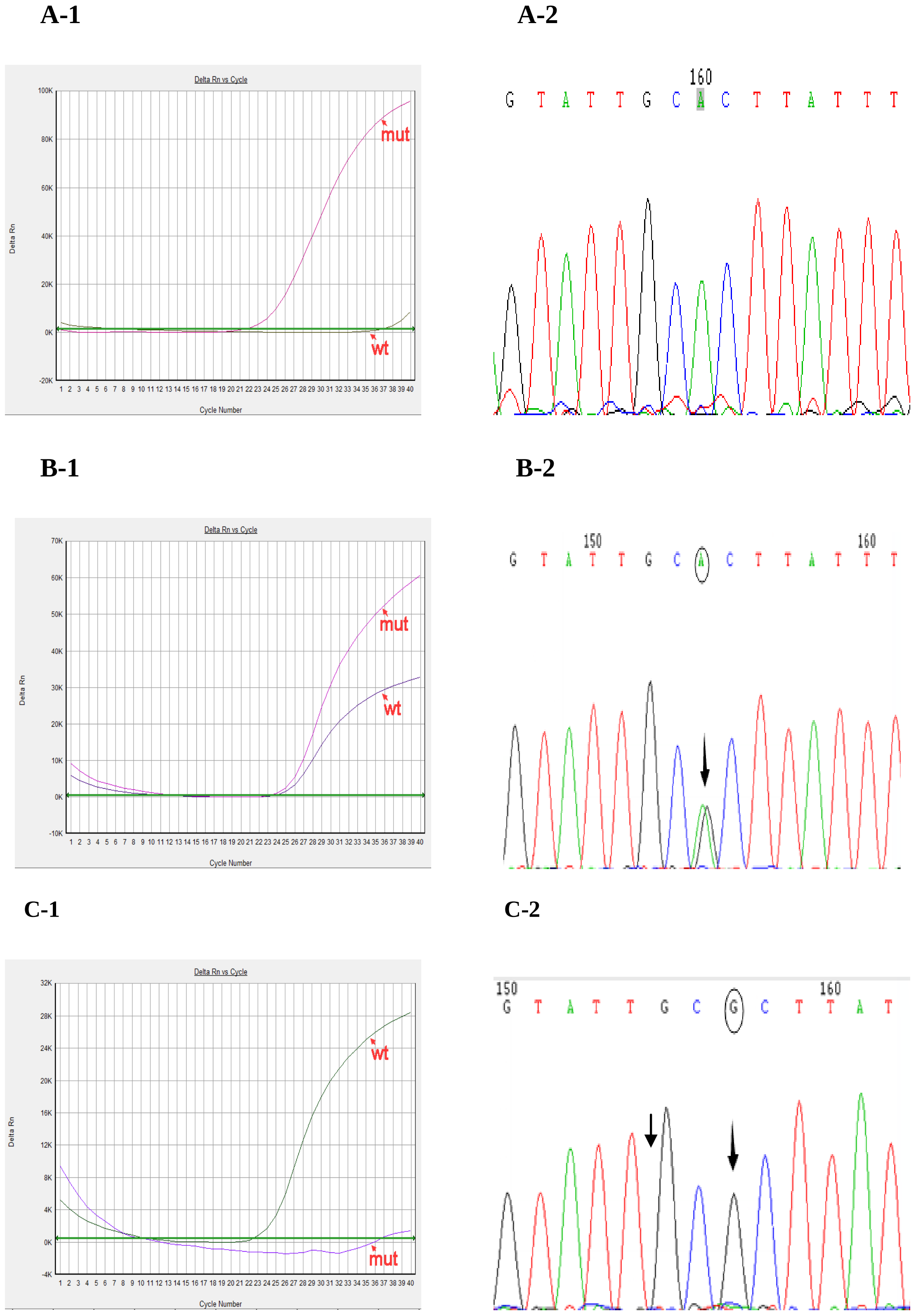

Figure9. ARMS-PCR result and Sequencing result diagram of c.158G>A site.

（A-1）:ARMS-PCR results of homozygous mutant samples; （A-2）:Sequencing results of homozygous mutant samples;（B-1）ARMS-PCR results of heterozygous mutant samples; （B-2）Sequencing results of heterozygous mutant samples （C-1）:ARMS-PCR results of wild-type samples;（C-2）:Sequencing results of wild-type samples. ↓:indicates a mutation site.

Supply8.

A B

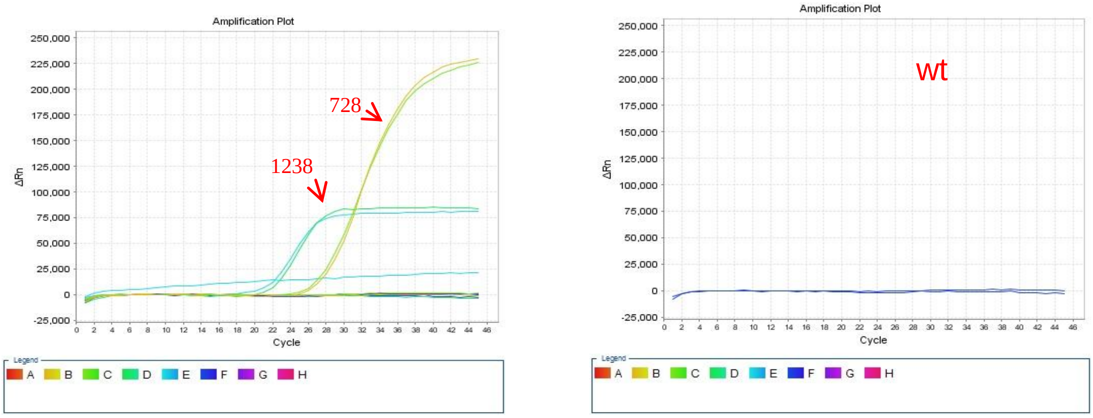

Figure1. ARMS-PCR results of c.728G>A+c.1238G>C site.

(A）Graph of positive sample amplification results （B）Results of wild-type sample amplification↓ : indicates a mutation site

A B

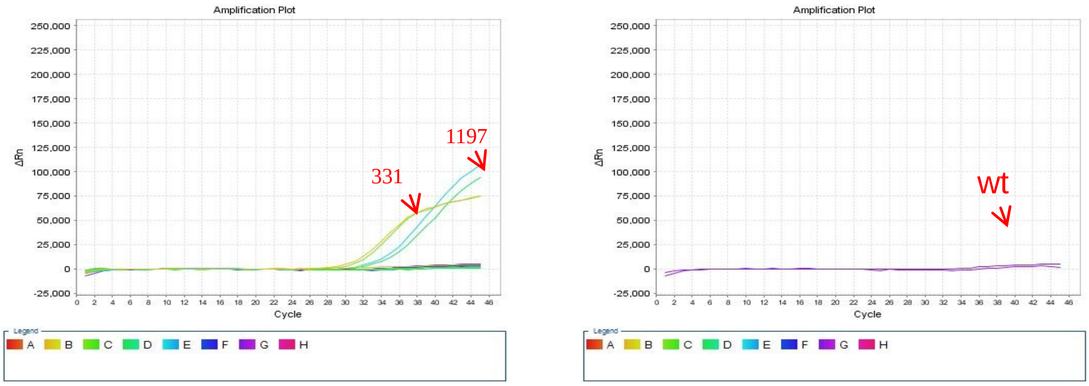

Figure 2. ARMS-PCR results of c.331C>T+c.1197A>T site

（A）Graph of positive sample amplification results. （B）Results of wild-type sample amplification. ↓: indicates a mutation site.

A B

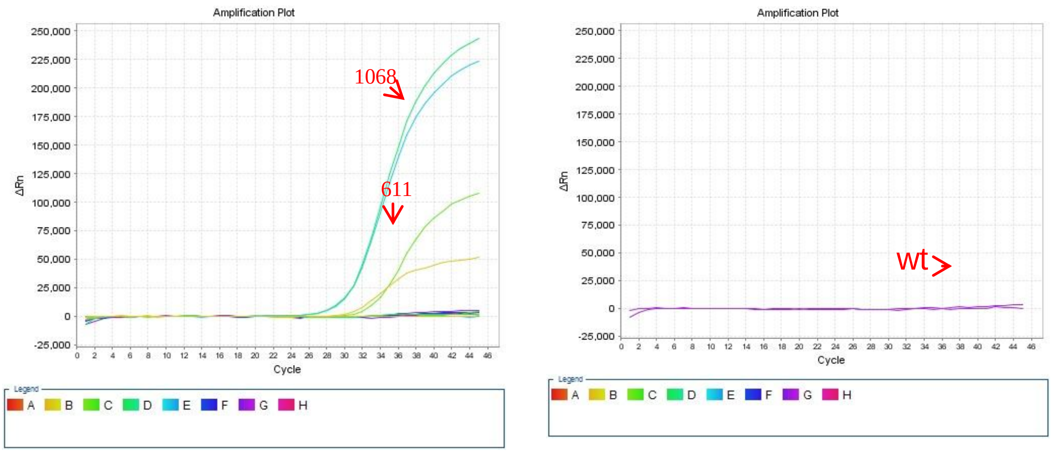

Figure 3. ARMS-PCR results of c.611A>G+c.1068C>A site

（A）Graph of positive sample amplification results （B）Results of wild-type sample amplification ↓: indicates a mutation site.

A B

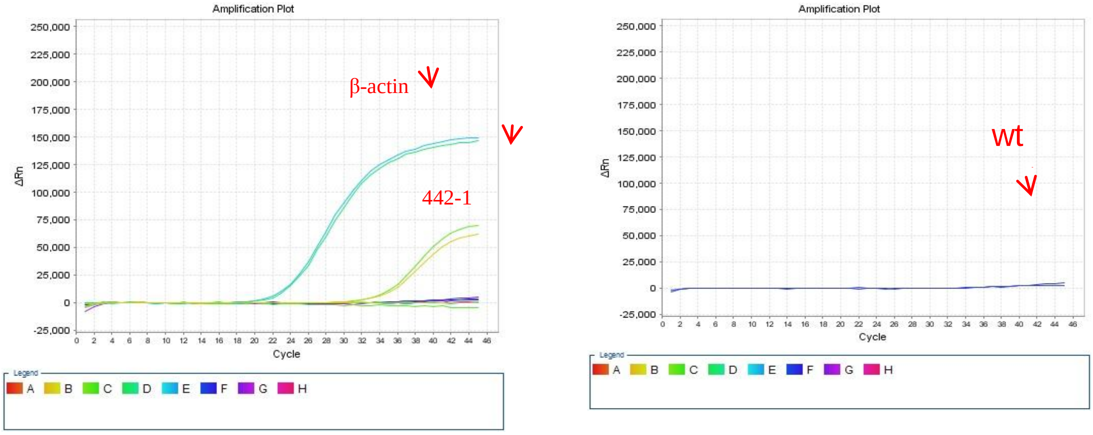

Figure4. ARMS-PCR results of c.442-1G>A+β-actin site.

（A）:Graph of positive sample amplification results （B）:Results of wild-type sample amplification↓: indicates a mutation site.

A B

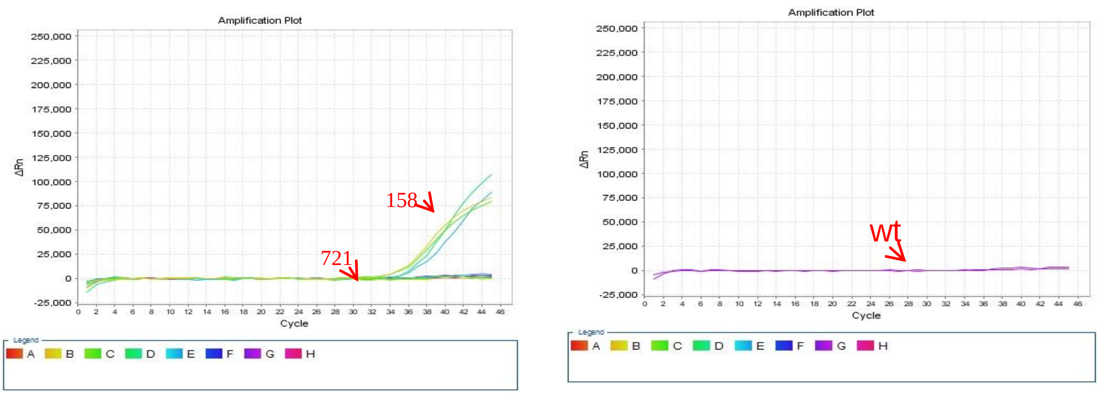

Figure5. ARMS-PCR results of c.721C>T+c.158G>A site.

（A）:Graph of positive sample amplification results. （B）:Results of wild-type sample amplification. ↓: indicates a mutation site.

Supply9.

A B

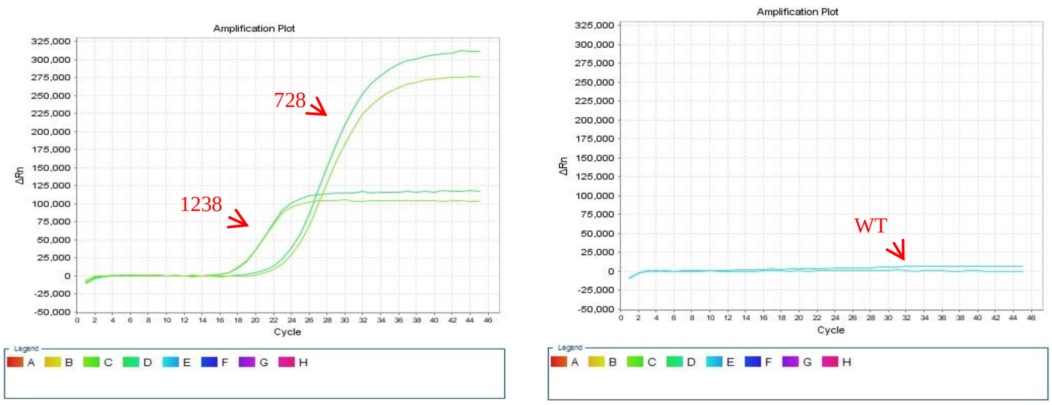

Figure1. ARMS-PCR results of c.728G>A+c.1238G>C site.

(A) Graph of positive sample amplification results (B) Results of wild-type sample amplification↓ : indicates a mutation site

A B

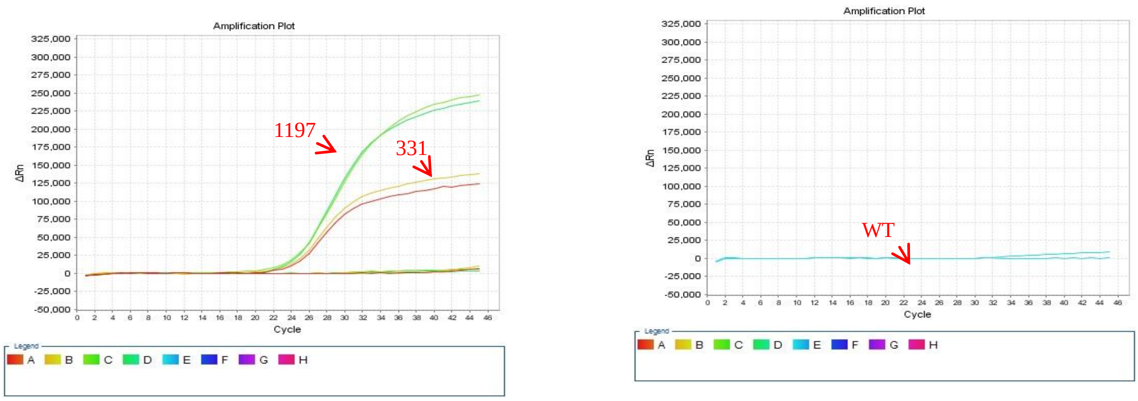

Figure 2. ARMS-PCR results of c.331C>T+c.1197A>T site

(A) Graph of positive sample amplification results. (B) Results of wild-type sample amplification. ↓: indicates a mutation site.

A B

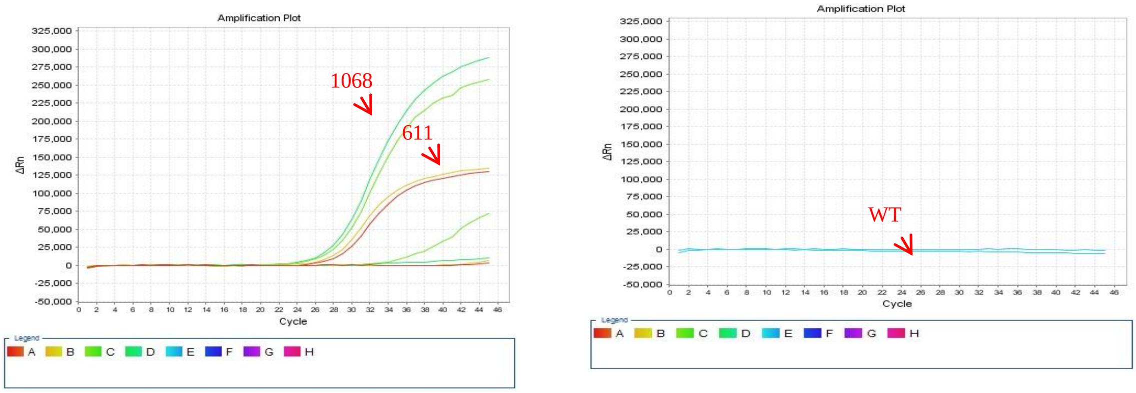

Figure 3. ARMS-PCR results of c.611A>G+c.1068C>A site

（A）Graph of positive sample amplification results （B）Results of wild-type sample amplification ↓: indicates a mutation site.

A B

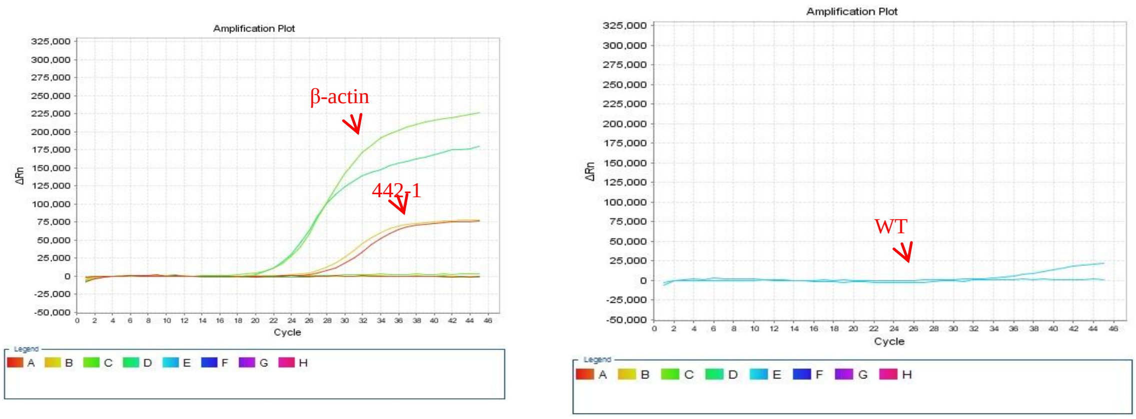

Figure4. ARMS-PCR results of c.442-1G>A+β-actin site.

（A）:Graph of positive sample amplification results （B）:Results of wild-type sample amplification↓: indicates a mutation site.

A B

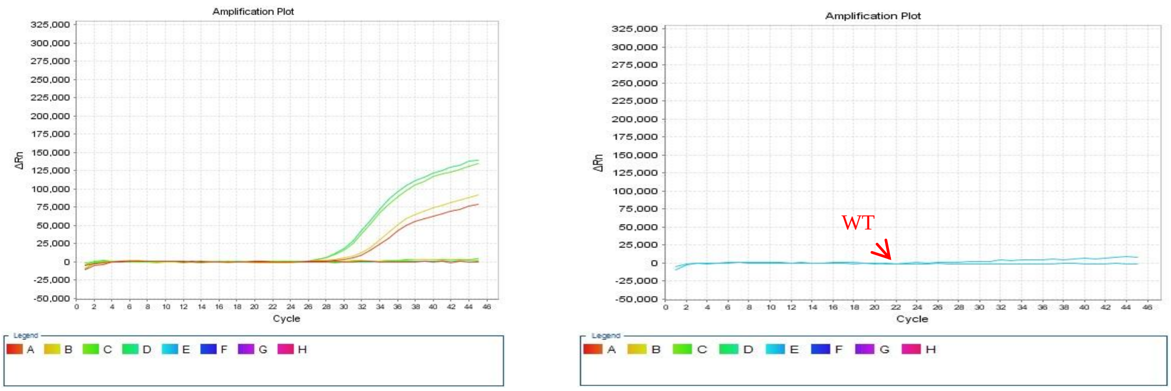

Figure5. ARMS-PCR results of c.721C>T+c.158G>A site.

（A）:Graph of positive sample amplification results. （B）:Results of wild-type sample amplification. ↓: indicates a mutation site.
